# Analysis of the methamphetamine-related deaths in Eastern Saxony, Germany, between 2005 and 2019

**DOI:** 10.1007/s12024-025-01023-6

**Published:** 2025-05-19

**Authors:** Henri Masing, Jörg Pietsch

**Affiliations:** Institute of Legal Medicine, Technische Universität Dresden, Fetscherstr. 74, D-01307 Dresden, Germany

**Keywords:** Methamphetamine, Cause of death, Circumstances, Epidemiology, Toxicology

## Abstract

Purpose: This study aims to evaluate the fatal consequences of the rising methamphetamine (MA) abuse in Eastern Saxony, Germany, and to examine the broader societal impact of MA on the region. Methods: Sociodemographic, forensic toxicological, forensic medical, and psychiatric data from 74 cases of deaths associated with MA were analyzed to identify significant trends and findings (2005–2019). Results: The majority of cases involved male individuals and non-natural deaths, with accidental MA intoxications and suicides being the predominant categories. The Years of Potential Life Lost (YPLL) was calculated at 49.8 years. The majority of natural deaths were related to cardiovascular conditions (e.g. cardiogenic shock, left ventricular hypertrophy (LVH), myocardial infarction), metabolic (e.g. diabetic ketoacidosis, alcohol withdrawal delirium) and inflammatory causes. In cases of fatal MA mono-intoxications and mixed intoxications involving MA, significantly higher MA blood concentrations were observed compared to intoxications primarily caused by other substances. Individuals in socioeconomically precarious situations are especially vulnerable to MA-associated deaths. Conclusion: There has been an increase in MA-related fatalities in Eastern Saxony since 2005. Accidental MA intoxications were the leading cause of death, followed by violent suicides. Challenging living conditions and social circumstances are especially vulnerable to MA-associated deaths. The study underlines the need for a comprehensive, coordinated approach to tackle the MA problem and reduce the number of MA-associated deaths.

## Introduction

The Free State of Saxony, located on the German-Czech border, is particularly affected by methamphetamine (MA, “Crystal”) abuse. Over the past 20 years, there has been a significant and consistently high level of both drug-related offenses and the quantity of MA seized by customs and police [1, 2]. Moreover, MA consumption in the region has notably increased until 2010 and remained stable at the high level since then [3]. Since 2005, the Institute of Legal Medicine in Dresden has recorded deaths linked to MA in autopsy cases, which remains the most frequent cause of drug-related fatalities in the region [4].

Like all stimulants, MA induces a characteristic symptomatology due to heightened sympathetic nervous system activity. This includes mydriasis, excessive sweating, hypertension, and tachycardia [5–7]. Chronic MA consumption is associated with significant cardiovascular, neurological, and venereological risks, and it exerts both cardiotoxic and neurotoxic effects at the cellular level [8–13].

At toxic doses, MA can cause life-threatening conditions such as hyperthermia with rhabdomyolysis, disruption of the blood-brain barrier (BBB), and brain edema, which may lead to multi-organ failure [5, 6, 14, 15]. Literature suggests that many, potentially even the majority, of deaths related to MA could be traumatic in nature, primarily resulting from accidents and suicides [16–20].

Globally, an increase in MA-associated fatalities has been observed since the turn of the millennium [7, 18, 21–30], with similar patterns emerging in Eastern Saxony.

This study aims to provide a comprehensive examination of the fatal effects of MA abuse in Eastern Saxony, using a case series of MA-related deaths, which is unparalleled in Germany to date. The study will assess these fatalities from sociodemographic, forensic, and toxicological perspectives, offering new insights into the impact of MA on society in the region.

This research seeks to clarify the rising incidence of MA-related deaths in Eastern Saxony and to evaluate the societal and public health implications. By analyzing forensic and toxicological data from these cases, the study aims to provide a clearer understanding of the lethal consequences of increasing MA abuse, offering a significant contribution to existing research in the field.

## Materials and methods

### Data collection

This study represents a retrospective epidemiological analysis of 6,148 autopsies conducted at the Institute of Legal Medicine in Dresden between 2005 and 2019. The data for the study were derived from autopsy reports, toxicological findings, and police investigation files.

A total of 74 drug-related deaths, in which methamphetamine was detected in the blood, were identified within the administrative district of Eastern Saxony, Germany. The following parameters were extracted from the autopsy reports: year and month of death, age, sex, weight, height, heart weight, results of histological examinations, manner of death, circumstances of death, primary cause of violence, route of methamphetamine administration (if applicable), and the stated cause of death.

Toxicological reports provided information on the concentrations of illegal drugs (amphetamines, THC, cocaine, opiates) and co-consumed substances (alcohol, medications). Additional data extracted from police investigation files included marital status, children, occupation, location of discovery, place of residence, psychiatric history, substance use history, and comorbid conditions.

### Forensic autopsy

All autopsies included in this study were performed at the Institute of Legal Medicine in Dresden, following orders from the public prosecutor’s offices. The determination of the cause of death was based on morphological and histological examinations. For the purposes of this study, parameters such as left ventricular cardiac hypertrophy (LVH) and heart weight were specifically analyzed for relevance.

### Toxicological analysis

In the context of toxicological and chemical analysis, blood, urine, and organ samples (if available) were tested for ethanol, illegal drugs, and medications. Screening procedures (immunoassays) were used alongside confirmatory chromatographic single-substance analyses, including gas chromatography-mass spectrometry (GC/MS), high-performance liquid chromatography with photodiode array detection (HPLC/PDA), and liquid chromatography-tandem mass spectrometry (HPLC/MS/MS).

Ethanol concentrations were determined using two independent GC/FID methods. The analysis of drugs of abuse (amphetamines, cannabinoids, cocaine, opiates) was carried out after extraction and derivatization using GC/MS. Quantitative detection of therapeutic agents was performed following liquid extraction, using HPLC/PDA and HPLC/MS/MS.

### Statistical analysis

Data collection and the creation of the data tables were performed using Microsoft Excel©. For statistical analysis, the software programs R Statistics and Prism 9 GraphPad© were utilized. Given the presence of numerous outliers in the methamphetamine serum concentrations the median was reported. A *p*-value of less than 0.05 was considered statistically significant. To assess statistical significance, the Student’s t-test and Mann-Whitney U test were applied.

### Classification of the MA-associated deaths

**Fig. 1:**
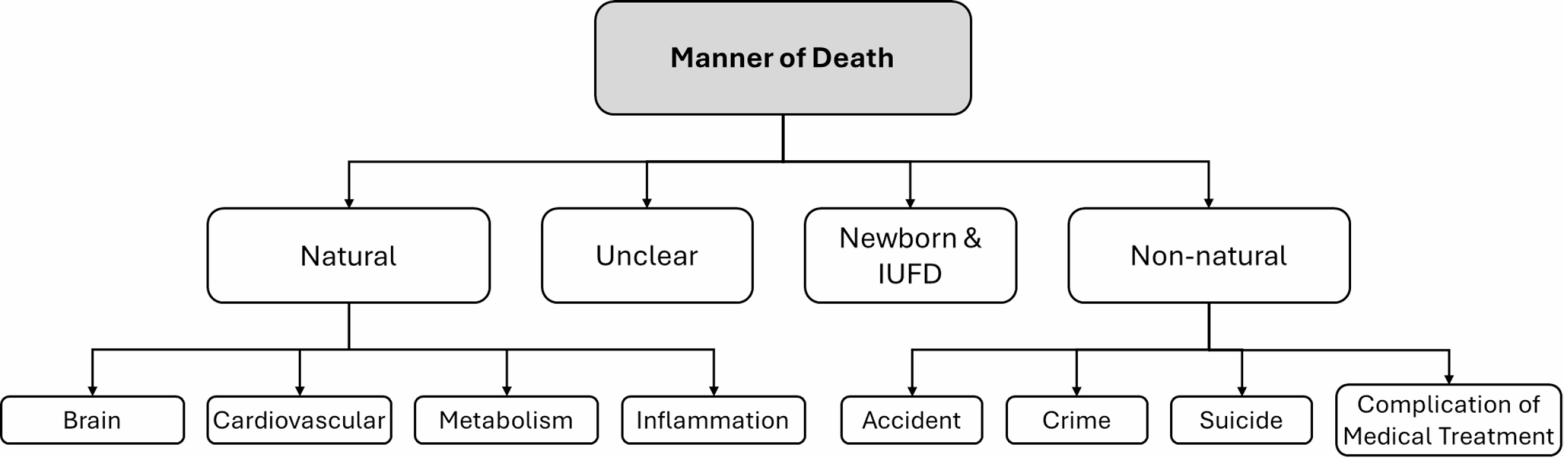
Classification of the MA-associated deaths

The manner of death was categorized into the following parameters: natural, non-natural, unclear and newborns or intrauterine fetal demise (IUFD) (Fig. 1).

## Results

Of the 6,148 autopsies conducted at the Institute of Legal Medicine Dresden between 2005 and 2019, a total of 74 cases were eligible for inclusion in the study. The clinical information is summarized annually in Table 1. There were 53 males and 21 females of mean age 29.59 ± 10.39 years. The manner of death for 14 cases (18.9%) was classified as natural, 54 cases (73.0%) as non-natural, 2 cases (2.7%) as unclear and 4 cases (5.4%) involved newborns or IUFD.

**Table 1:** Characteristics of MA-associated deaths in Eastern Saxony, 2005–2019

Year of Death	*n*	Age (a)	Sex		Manner of Death			
			Male	Female	Natural	Non- natural	Unclear	Newborn & IUFD
2005	4	31.25 ± 12.76	3 (75.0)	1 (25.0)	2 (50.0)	2 (50.0)	0	0
2006	1	26.00 ± 0.00	1 (100)	0	0	1 (100)	0	0
2007	4	22.75 ± 18.66	2 (50.0)	2 (50.0)	0	3 (75.0)	0	1 (25.0)
2008	3	28.00 ± 4.36	2 (66.7)	1 (33.3)	2 (66.7)	1 (33.3)	0	0
2009	1	23.00 ± 0.00	1 (100)	0	0	1 (100)	0	0
2010	6	24.83 ± 3.87	5 (83.3)	1 (16.7)	0	6 (100)	0	0
2011	9	27.11 ± 3.76	5 (55.6)	4 (44.4)	1 (11.1)	8 (88.9)	0	0
2012	2	27.00 ± 1.41	2 (100)	0	0	2 (100)	0	0
2013	6	27.67 ± 7.8	5 (83.3)	1 (16.7)	2 (33.3)	4 (66.7)	0	0
2014	3	39.33 ± 8.74	2 (66.7)	1 (33.3)	0	3 (100)	0	0
2015	12	27.17 ± 14.01	8 (66.7)	4 (33.3)	4 (33.3)	6 (50.0)	0	2 (16.7)
2016	5	28.40 ± 6.5	4 (80.0)	1 (20.0)	1 (20.0)	4 (80.0)	0	0
2017	3	38.67 ± 1.53	2 (66.7)	1 (33.3)	0	3 (100)	0	0
2018	6	37.50 ± 3.56	4 (66.7)	2 (33.3)	1 (16.7)	4 (66.7)	1 (16.7)	0
2019	9	33.44 ± 14.37	7 (77.8)	2 (22.2)	1 (11.1)	6 (66.7)	1 (11.1)	1 (11.1)
**Total**	**74**	**29.59 ± 10.39**	**53 (71.6)**	**21 (28.4)**	**14 (18.9)**	**54 (73.0)**	**2 (2.7)**	**4 (5.4)**

Values are presented as mean ± standard deviation (age) or number (%, sex, manner of death)

IUFD = Intrauterine Fetal Demise

The Years of Potential Life Lost (YPLL) for the examined autopsy cases was 49.8 years. For males, the YPLL was 47.1 years, while for females, it was 53.0 years. A significant proportion of the deceased were unemployed (42.6%). Additionally, the majority of the deceased individuals resided alone. The bodies were discovered in various locations, including their residences (the majority), public places, medical facilities, and correctional institutions. Notably, more men than women were found deceased in outdoor settings. Most of the deceased individuals were single (51.5%), with smaller proportions being in partnerships (13.2%), married (2.9%), or divorced (2.9%). In 29.4% of the cases, marital status was not recorded. Only 10.3% of the deceased had children. Among those with children, the average age was 37.1 years, compared to 30.5 years for those without children. All individuals with children were found at home. A significant proportion (71.4%) of the deceased persons with children had committed suicide, primarily by hanging.

A total of six cases were excluded from the study. Specifically, two cases were excluded due to the failure to determine the cause of death (unclear), and four cases involved newborns or IUFD, which were deemed not comparable and thus excluded.

### Evaluation of autopsy findings based on manner of death

Table 2 categorizes 68 autopsy cases into natural (*n* = 14) and non-natural (*n* = 54) manners of death, detailing the major pathological findings in each group.

**Table 2:** Evaluation of autopsy findings based on manner of death

Manner of Death		Major Autopsy Findings
**Natural** (***n*** = **14**)	Brain (*n* = 1)	Brain tissue softening with intracranial bleeding (1)
	Cardiovascular (*n* = 6)	Cardiogenic shock after LVH (1)
		Small bowel infarction after colon cancer (1)
		Acute left ventricular failure after hypertension (1)
		Acute left ventricular failure after coronary athero-sclerosis (1)
		Mycardial infarction (1)
		Acute coronary insufficiency with coronary heart disease and COPD (1)
	Metabolism (*n* = 3)	Ketoacidosis due to Type I Diabetes (2)
		Alcohol withdrawal delirium (1)
	Inflammation (*n* = 4)	Septic encephalitis after myocarditis (1)
		Pneumonia (2)
		Myocarditis (1)
**Non-natural** (***n*** = **54**)	Accident (*n* = 29)	Intoxication (23)
		Polytrauma (traffic accident) (3)
		Subarachnoid hemorrhage (1)
		Hemorrhagic shock (traffic accident) (1)
		Drowing (1)
	Crime (*n* = 4)	Gunshot wound, heart involvement (1)
		Hemorrhagic shock, stab wounds (2)
		Strangulation (1)
	Suicide (*n* = 20)	Hemorrhagic shock (2)
		Hanging (11)
		Poytrauma, fall from a height (6)
		Intoxication (1)
	Complication of medical treatment (*n* = 1)	Hypoxic brain damage after misplaced tube (1)

The majority of natural deaths were related to cardiovascular conditions (*n* = 6), including cardiogenic shock following LVH, myocardial infarction, and various forms of acute heart failure. Metabolic causes were also significant (*n* = 3), notably diabetic ketoacidosis and one case of alcohol withdrawal delirium. Inflammatory causes (*n* = 4) included septic encephalitis secondary to myocarditis, two cases of pneumonia, and one case of isolated myocarditis. A single case involved neurological findings, specifically brain tissue softening with intracranial bleeding.

As for non-natural deaths, accidental deaths were the most frequent (*n* = 29), with the majority due to intoxication (23 cases). Suicides accounted for 20 cases, with hanging being the most common method (11 cases), followed by fatal falls resulting in polytrauma (6). Criminal deaths (*n* = 4) included fatal gunshot, stab wounds and one case of strangulation. There was also one case classified as a complication of medical treatment, involving hypoxic brain damage due to a misplaced tube (Table 2).

The analysis reveals in this cohort that non-natural deaths significantly outnumber natural deaths, with intoxication and suicide (especially by hanging or falling) as predominant causes. Among natural deaths, cardiovascular and inflammatory diseases were the leading contributors. This distribution highlights the importance of forensic investigations in uncovering the precise cause of death, especially in non-natural cases.

### MA blood concentration in cases with intoxication and non-intoxication

The determined MA blood concentrations, divided according to MA mono-intoxication (group A), MA intoxication with co-consumption (group B), not MA-related intoxication (group C) and death due to other cases than intoxication in general (group D), are summarized in Table 3.

**Table 3:** MA blood concentrations, divided in groups A - D

Group	*n*	MA (µg/ml)
A	12	8.65 (0.54–26.90)
B	4	2.92 (0.70–4.06)
C	8	0.12 (0.05–0.54)
D	44	0.18 (0.02–4.49)

Values are presented as median (range)

MA = Methamphetamin

Within the groups of intoxications, MA mono-intoxications (A) and MA poly-intoxications (B) exhibit significantly higher MA concentrations compared to cases where death was not related to MA intoxication (C and D).

### Left ventricular cardiac hypertrophy

In 13 cases (18.6%) an autopsy revealed LVH with a wall thickness greater than 12 millimeters in the left outflow tract. Of these, eleven cases were male and two were female.

42.9% of natural deaths exhibited LVH, in contrast to 12.5% of unnatural deaths. Significantly more natural deaths in the examined cases showed LVH in the autopsy results (*p* = 0.031).

### Heart weight

The heart weight was determined in 66 cases. Natural deaths (*n* = 14, 401.4 g ± 82.6 g) exhibit a significantly higher heart weight (*p* = 0.020) compared to unnatural deaths (*n* = 52, 352.9 g ± 62.7 g).

### Pre-existing psychiatric illness

In 15 cases (21.4%) a known psychiatric disorder was identified. Among these, 53.3% had depression, 40.0% had psychoses, and 6.7% had attention-deficit/ hyperactivity disorder (ADHD). The MA serum concentration in cases with a known pre-existing psychiatric illness ranged from 0.03 µg/ml to 26.9 µg/ml, with a median of 0.47 µg/ml and an average concentration of 3.08 ± 6.83 µg/ml. Individuals with pre-existing psychiatric conditions almost exclusively died from violent causes.

## Discussion

In this study, 74 cases of MA-associated deaths, investigated between 2005 and 2019 in Eastern Saxony, Germany, were evaluated. According to data published from Norway [21], this study is the first to address MA-related deaths in Europe.

Consistent with the majority of published papers on MA-associated deaths the deceased in Saxony were predominantly male (71.6%, Table 1). Interestingly, the deceased were significantly younger at the time of death (mean 29.59 years, Table 1) than in other studies [18, 27, 29]. No trend can be derived from the annual distribution of MA-related deaths in Saxony. The number of cases ranged from just one (2006, 2009) to twelve cases in 2015 (Table 1). Possibly, a significant number of MA-related deaths may remain unreported, contributing to a “dark field” of undetected fatalities [31].

The largest group of non-natural methamphetamine (MA)-associated deaths in this study was the accident group, with intoxication being the primary cause of these accidents (Table 2). This finding is consistent with general literature on MA-related deaths [18, 27, 29, 32, 33]. Direct MA intoxication was the cause of death in 17.6% (*n* = 12) of all cases, including two cases with toxic MA blood concentrations and seven cases with comatose-lethal levels of MA. In the remaining cases, MA was present only as a toxicological finding, a pattern also observed in studies from Taiwan [33, 34], Japan [27], Iran [22] and Australia [18, 29, 32].

Suicides were the second most frequent cause of death following accidents (Table 2). The methods of suicide were predominantly violent, such as stab wounds to the body cavities, falls from height, and hanging, aligning with findings in the existing literature [18, 22, 27, 29, 32, 34]. Individuals with pathological consumption patterns of MA are at an elevated risk of suicide, particularly if they exhibit risk factors such as a Beck Depression Inventory score > 20 [35, 36], female gender, intravenous use, a history of severe depression, or previous inpatient treatment for psychiatric illness [18, 25, 29, 33, 34].

Criminal cases in the study involved victims of violent acts. The average MA blood concentration in these cases was in the lower range of MA exposure, supporting the theory that the victims were not the direct cause of their deaths due to MA but were rather victims of other violent acts. Notably, 75% (*n* = 3) of the intrauterine fetal demise (IUFD) observed in this cohort were categorized within this group.

The observation that significantly fewer natural deaths (Table 2) were recorded overall, and that the majority of natural deaths were due to cardiovascular causes (manifesting as LVH and increased heart weights), is consistent with findings in the literature [18, 22, 27, 29, 32, 33]. It is presumed that metabolic disorders and infections, common in chronic MA users, may have contributed to these natural deaths. These conditions include general neglect, poor management of chronic diseases like diabetes, intravenous drug abuse, and a weakened immune system, which predispose individuals to fatal conditions such as pneumonia, septic myocarditis, and encephalitis.

This study’s unique number of MA-associated deaths offers a more nuanced view of the relationship between MA intoxication and related fatalities. In contrast to the numerous studies on MA-related deaths, the present study also determined the MA blood concentration in the blood of the deceased. When compared to reference values starting at 0.20 µg/ml [37, 38] and toxic concentrations noted in the literature starting at ca. 0.50 µg/ml [18, 27, 37–40], the median lethal blood concentration measured in this study (8.65 µg/ml, Table 3) was ca. 17 times higher. During autopsy, all cases exhibited nonspecific signs of intoxication, including dilated hollow organs, liquid postmortem blood, pronounced brain and lung edema, acute congestion of parenchymal organs, and the presence of Tardieu spots. The median MA blood concentration in mixed intoxications (2.92 µg/ml, Table 3) was nearly six times higher than the toxic concentrations described in the literature [27, 37–40].

Darke et al. [18] and Stronach et al. [29] found that over approx. 40% of cases involved multiple substances, and this study similarly found that approx. 50% of cases had consumed both MA and at least one other drug. While Eastern Saxony exhibited fewer cases of poly-intoxication, the substances involved were similar to those reported internationally, including opiates, alcohol, antidepressants, and antipsychotic drugs [18, 22, 30, 32]. A direct comparison of autopsy material revealed that although the number of accidental drug intoxications was lower, MA intoxications were more prevalent in Eastern Saxony than in Australia [18, 29].

The results of this study, alongside comparable international research, highlight the urgent need for action to address MA use in Eastern Saxony. These findings reveal a pattern of precarious living conditions and social circumstances among the deceased, including high unemployment rates, singlehood, and low rates of parenthood. These factors may contribute to an elevated risk of MA-related fatalities [17, 24, 26, 33].

While MA still plays a relatively minor role in Germany overall, some countries worldwide are already severely affected. Individuals in socioeconomically precarious situations are especially vulnerable to MA-associated deaths [18, 22, 33]. To reduce this, preventive measures such as training programs aimed that professionals and the public can help raise awareness of the risks associated with MA use. Increased cooperation between law enforcement agencies, including the Federal Criminal Police Office and State Criminal Police Offices, is critical for combating the illegal drug trade and controlling the spread of MA, particularly in prisons. Furthermore, MA should be listed as a separate drug in official government datasets, rather than being grouped under stimulants or together with amphetamines in publications such as Police Crime Statistics. In addition, it appears essential that efforts be approached to reduce the “dark field” of unreported MA-related deaths.

Emergency medical personnel and rescue services play a crucial role in responding to MA-related incidents. Enhanced training for these professionals would enable them to recognize symptoms of MA intoxication more rapidly, improving their responses to acute emergencies and the accurate documentation of deaths [31]. Additionally, the implementation of specific ICD-10 coding for MA could aid in better tracking and reporting of MA-associated deaths. Currently, there is only one code (F15) for all stimulants, which complicates the recording of MA-related fatalities and hinders the development of targeted preventive measures.

In conclusion, as Stronach et al. formulated for Australia [29], there is also a need for screening and early management approaches in Germany. A first step could be an increased service level integration of mental health and drug treatment services. This study provides valuable insights into the impact of methamphetamine on public health in Eastern Saxony, Germany, and underlines the need for a comprehensive, coordinated approach to tackle the problem and reduce the number of deaths caused by MA.

### Limitations

While this study provides valuable insights, there are several limitations to be considered, both within the context of this research and in comparison to other studies:

- Geographic Limitations: The catchment area of this study is restricted to Eastern Saxony, which may limit the generalizability of the findings to other regions in Germany or internationally.
- Sample Size: Although the case cohort is unique within Germany to date, it remains relatively small, which may impact the statistical power and the ability to draw broader conclusions.
- Incomplete Sociodemographic Data: The dataset often lacks complete sociodemographic information, which could provide a more comprehensive understanding of the individuals involved and potentially identify additional risk factors or trends.
- Unspecified Consumption Patterns: The form of methamphetamine consumption is frequently not specified in the data, which limits the ability.
- to explore its role in the outcomes observed. There is a significant degree of heterogeneity in the methods of consumption, which complicates the analysis.
- ICD-10 Coding Issues: The ICD-10 coding system, where all stimulants are grouped under the F15 code, does not differentiate between methamphetamine and other amphetamines. This can lead to misclassification in data reporting and make it difficult to track MA-specific fatalities in official records.
- Postmortem Examination Limitations: Postmortem investigations are a significant limitation, as the “dark field” for drug-related and non-natural deaths is considerably larger than what is currently known or reported [31]. This means that the actual number of MA-associated deaths may be underreported, leading to potential underestimation of the problem.
- These limitations should be taken into account when interpreting the results and when considering the broader implications for public health and policy.

## Conclusions

In summary, there has been an increase in MA-related fatalities in Eastern Saxony since 2005. Accidental MA intoxications were the leading cause of death, followed by violent suicides. Challenging living conditions and social circumstances are especially vulnerable to MA-associated deaths. The study underlines the need for a comprehensive, coordinated approach to tackle the MA problem and reduce the number of MA-associated deaths.

### Points

1. MA-associated deaths are of significant concern in Eastern Saxony, Germany.
2. Accidental MA intoxication is the leading cause of death, followed by violent suicides.
3. The strongest risk factors for MA-associated deaths include female gender, intravenous use, a history of severe depression, and previous inpatient treatment for psychiatric illness.
4. The deceased often exhibited high unemployment rates, singlehood, and low rates of parenthood, reflecting their challenging living conditions and social circumstances.
5. A coordinated, multi-faceted approach is essential to address and reduce MA-related fatalities in Eastern Saxony, Germany.
